# Robust Heart Rate Variability Measurement from Facial Videos

**DOI:** 10.3390/bioengineering10070851

**Published:** 2023-07-18

**Authors:** Ismoil Odinaev, Kwan Long Wong, Jing Wei Chin, Raghav Goyal, Tsz Tai Chan, Richard H. Y. So

**Affiliations:** 1 PanopticAI Ltd., Hong Kong, China; kylewong@panoptic.ai (K.L.W.); jwchin@connect.ust.hk (J.W.C.); rgoyalaa@connect.ust.hk (R.G.); ttchanac@connect.ust.hk (T.T.C.); rhyso@ust.hk (R.H.Y.S.); 2 Department of Chemical and Biological Engineering, Hong Kong University of Science and Technology, Hong Kong, China; 3 Department of Computer Science and Engineering, Hong Kong University of Science and Technology, Hong Kong, China

**Keywords:** heart rate variability, remote photoplethysmography, wavelet scattering transform, RMSSD, SDNN, Baevsky stress index

## Abstract

Remote Photoplethysmography (rPPG) is a contactless method that enables the detection of various physiological signals from facial videos. rPPG utilizes a digital camera to detect subtle changes in skin color to measure vital signs such as heart rate variability (HRV), an important biomarker related to the autonomous nervous system. This paper presents a novel contactless HRV extraction algorithm, WaveHRV, based on the Wavelet Scattering Transform technique, followed by adaptive bandpass filtering and inter-beat-interval (IBI) analysis. Furthermore, a novel method is introduced to preprocess noisy contact-based PPG signals. WaveHRV is bench-marked against existing algorithms and public datasets. Our results show that WaveHRV is promising and achieves the lowest mean absolute error (MAE) of 10.5 ms and 6.15 ms for RMSSD and SDNN on the UBFCrPPG dataset.

## 1. Introduction

Heart rate variability is the variation in time between consecutive heartbeats. It is closely related to the autonomous nervous system (ANS), actual heart sound, blood pressure, and mental well-being [1]. Traditionally, HRV has been measured using a contact-based electrocardiogram (ECG), which may cause some patients to feel uncomfortable because it requires attaching electrodes to various parts of the body. Recently, non-contact measurement of HRV has gained momentum due to its user-friendly nature and suitability. Contactless HRV can be obtained from an optical technique known as remote plethysmography (rPPG) by using an off-shelf digital camera.

In recent years, there has been a growing interest in heart rate variability (HRV) estimation using remote photoplethysmography (rPPG), and many researchers have focused on developing robust and accurate algorithms for this purpose. Typically, a pipeline for rPPG-based HRV estimation includes several stages, such as face detection and tracking, skin segmentation, region of interest (ROI) selection, and rPPG construction [2,3,4,5]. In addition, there are numerous post-processing steps that can be applied to clean, filter, or denoise the rPPG signal to improve the accuracy of HRV estimation.

One such study by Mitsuhashi et al. [6] obtained the rPPG signal from facial videos using the spatial subspace rotation (2SR) method [7]. 2SR is an algorithmic method that extracts a pulse signal by calculating the spatial subspace of skin pixels and measuring its temporal movement, and it does not require skin-tone priors. They subsequently applied detrending, heart-rate frequency bandpass filtering (0.75–3 Hz), interpolation, and valley detection to source HRV and estimate stress. Martinez-Delgado et al. [8] employed color amplification on the red channel and peak detection to calculate multiple time-domain and frequency-domain HRV metrics. Qiao et al. [9] utilized independent component analysis (ICA) to obtain the rPPG signal and subsequently applied detrending, normalization, and moving average filter to further clean and smooth the rPPG signal. Afterward, they acquired heart rate and time-domain HRV metrics by detecting the peaks of the cleaned rPPG signal. Li et al. [2] obtained the rPPG signal using a CHROM algorithm [10], a method that exploits color differences in RGB channels to eliminate specular reflection and reduce noise due to motion. Then, they proposed a post-processing denoising step called Slope Sum Function (SSF), which enhances the quality of the signal and facilitates peak detection by increasing the upward trend and decreasing the downward trend of the rPPG signal. Lastly, heat rate and time-domain HRV metrics were evaluated based on the peak detection results.

A wavelet-based approach was proposed by Huang et al. [3] and He et al. [4]. Huang et al. [3] sourced the rPPG signal by utilizing the CHROM method [10] and further added a post-processing step based on a continuous wavelet transform, termed CWT-BP and CWT-MAX. CWT-BP is defined as a bandpass filter (0.75–4 Hz), while CWT-MAX is a denoising step based on the scale of the CWT coefficients. During the CWT-MAX step, windows from the signal are chosen and coefficients that have the largest values within a particular window are selected to reconstruct the signal by inverse CWT. He et al. [4] further improved CWT-based denoising methods by introducing CWT-SNR, which selects coefficients based on the signal-to-noise ratio of the reconstructed rPPG signal. Both methods implemented peak-detection algorithms to acquire time-domain HRV metrics and heart rate.

In another research, Gudi et al. [5] sourced the rPPG signal by using the plane orthogonal to skin (POS) [11], a method that projects the pulsatile part of the RGB signal to the plane orthogonal to the skin thereby reducing specular and motion noise. Then they applied further motion noise suppression and narrow fixed bandpass filtering to clean the rPPG signal and subsequently extracted the HRV by detecting peaks and applying HRV formulae. They calculated both time-domain and frequency-domain metrics and benchmarked and tested their algorithm on numerous public datasets. Furthermore, they introduced a method to remove noise artifacts from ground truth PPG signals. In another study, Pai et al. [12] introduced a novel approach HRVCam. HRVCam applied signal-to-noise ratio (SNR) based adaptive bandpass filtering to the rPPG signal and then used a discrete energy separation algorithm (DESA) to calculate various frequency bands. These instantaneous frequencies are transformed to the time domain to evaluate time-domain HRV metrics. Overall, traditional methods have focused mostly on post-processing steps such as bandpass filtering, detrending, and continuous wavelet transform to clean noisy rPPG signals.

A deep learning approach was presented by Song et al. [13]. According to this approach, first, a candidate rPPG signal is calculated with traditional algorithmic methods such as CHROM [10]. Then, a generative adversarial network (GAN) is employed to filter out and denoise the signal by generating a cleaner version of that rPPG signal. An additional study by Yu et al. [14] proposed an end-to-end deep learning model to obtain an rPPG signal. Their model is based on different 3D-CNN and LSTM networks and benchmarked against heart rate and frequency-domain HRV metrics.

All listed HRV algorithms suffer from relatively poor results when compared with ground truth contact-based values. This may be due to limitations in the non-contact measurement techniques used by these algorithms, which can result in inaccuracies and lower overall performance. Additionally, deep learning models require a large amount of data to train on, which can be expensive. Since HRV is highly sensitive to noise, improved algorithms should be devised to decrease the gap between contact and camera HRV. Therefore, in this paper, we introduce the following:

1. A novel HRV algorithm, WaveHRV, based on the Wavelet Scattering Transform technique, followed by adaptive bandpass filtering and statistical analysis of inter-beat-intervals (IBIs);
2. Validation of our algorithm on various public datasets, which achieved promising results;
3. An innovative preprocessing step to filter out noisy ground truth data.

## 2. Method

**Figure 1:**
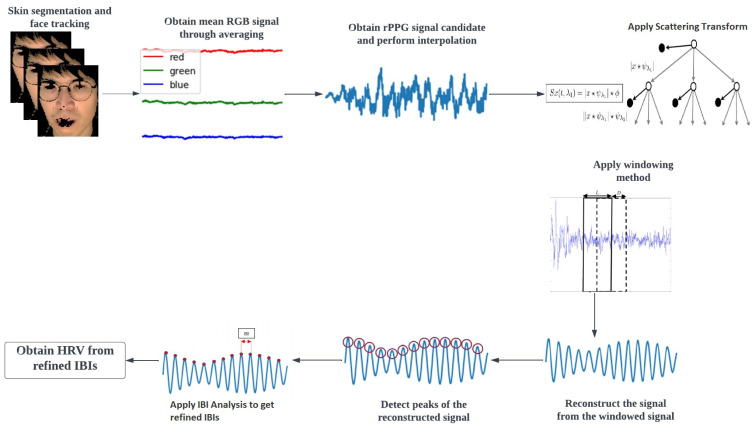
Pipeline to extract heart rate variability from facial videos.

The heart rate variability extraction pipeline from a video is presented in Figure 1. Initially, the subject’s face is detected and tracked over time by Medipipe FaceMesh [15]. This is followed by a process of skin segmentation to remove non-skin regions that would improve signal quality. Then, the meanRGB signal is acquired by taking the average of each frame spatially and concatenating them temporally. This meanRGB signal is fed to the plane orthogonal to skin (POS) [11] algorithm to get the rPPG signal candidate. POS is a robust method that projects the pulsatile part of the RGB signal to the plane orthogonal to the skin while employing division and multiplication of different channels to cancel out noise due to motion and other specular artifacts that are assumed to affect all color channels equally. The rPPG signal is interpolated to the nearest power of 2 framerate to make it easier to work with the scattering transform and make the signal spaced equally in time. Subsequently, scattering transform (Section 2.1), windowing method (Section 2.2), and IBI analysis (Section 2.3) are applied to obtain HRV from the interpolated rPPG signal.

### 2.1. Scattering Transform

The scattering transform (ST) [16] is a complex-valued convolutional neural network (CNN) whose filters are fixed wavelets that has modulus as non-linearity and averaging as pooling. It is invariant to translation, frequency shifting, and change in scale. The wavelet scattering transform can be constructed by taking a signal and passing it through a series of wavelet filters called filter banks and modulus non-linearity. Each wavelet within the filter bank is derived from a single wavelet by changing frequency and time. The output of each layer is then passed through another set of filter banks and modulus non-linearity, creating a hierarchical structure of representations. Each layer captures different levels of time and frequency information, with the first layer capturing the energy density of the frequencies over time. *Nth* order coefficients are given by

*S_N_*(*t*, *λ*_1_, …*λ_N_*) = |*r*(*t*) ∗ *ψ_λ_*_1_|… ∗ *ψ_λ__N_*| ∗ *ϕ* (1)

where *r*(*t*) is a signal, *ψ_λ_* is a wavelet of scale *λ*, *ϕ* is average pooling, |…| is complex-valued modulus operation and ∗ is convolutional operation. In this paper, the Kymatio Library [17] was used to implement scattering transform, and the Morlet wavelet was chosen to convolve with the signal, which is given by:

*ψ_w_*(*t*) = *Kπ*^−1/4^*e^iωt^e*^−*t*^2^/2^ (2)

where *K* is a normalization constant, *ω* is frequency, and *t* is time. Morlet wavelet has been previously employed in PPG research [18] because its Gaussian envelope ensures that the Morlet wavelet is localized in both time and frequency domains, making it suitable for analyzing signals with non-stationary and time-varying properties.

**Figure 2:**
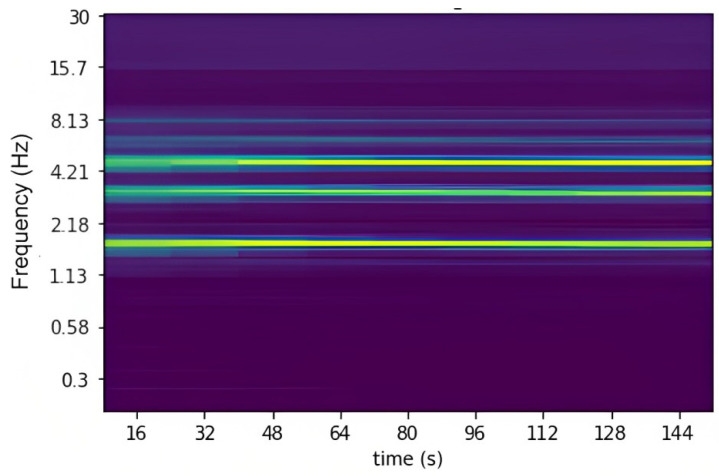
Example of the coefficients of the first-order scattering transform.

Lastly, an example of coefficients of first-order ST of a PPG signal with a pooling size of 16 s and filter bank of 20 is given in Figure 2. Frequencies in the y-axis increase exponentially, while time in the x-axis is given as discreet numbers that are multiples of 16 s due to chosen pooling size.

### 2.2. Windowing

The interpolated rPPG signal is first cleaned with Butterworth bandpass filtering of order 7 with band size 0.7–5 Hz to acquire *rPPG_clean_*. Then, the first-order scattering transform is applied to the obtained signal as explained in Section 2.1 with a pooling size of 16 s and filter bank of 20. The selection of the pooling size and number of wavelets within the filter bank is task dependent. In the context of our study, simulations revealed that higher frequency resolution generated more favorable outcomes than time resolution. Consequently, a pooling size of 16 s was deemed optimal as it represented a balance between time and frequency resolution. Furthermore, an augmented number of wavelets in the filter bank correlates with an increased frequency resolution. However, this may pose two challenges: firstly, higher computational costs, and secondly, increasing the number of wavelets in the filter bank usually enhances resolution in the higher frequency ranges that are beyond the heart rate region.

**Figure 3:**
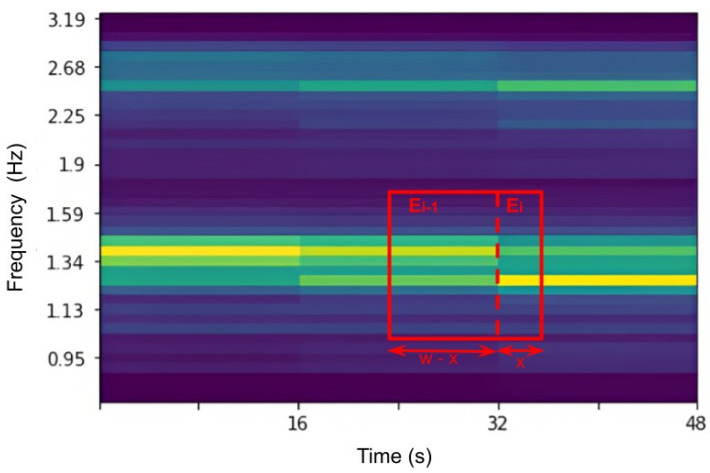
Example of the energy calculation for a particular window of the first-order scattering transform.

Afterward, a windowing step, shown in Figure 3, is applied on *rPPG_clean_* in the following manner:

1. For each window of size *w* calculate the energy around the first harmonic by the given equation:

(3) $$E=\frac{w-x}{w}E_{i-1}+\frac{x}{w}E_{i}$$

where *w* is window size, *E_i_* is the energy at time, *i*, and *x* is the difference between right end of the window and time *i*.

**Figure 4:**
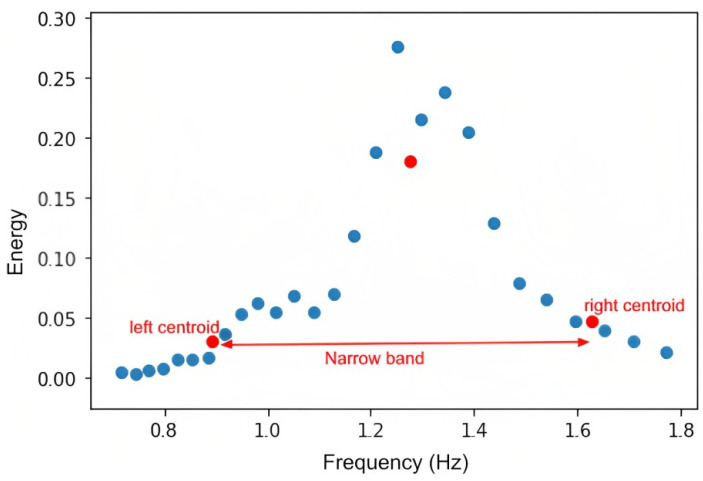
Narrow band calculation with K-Means Clustering (K = 3).

2. Construct K-Means (K = 3) clustering with frequency and energy, *E*, as an input and k-mean++ as a centroid initialization to obtain a narrow band, as shown in Figure 4. The centers of the clusters are shown in red in the Figure 4. Then, the band size is [left centroid, right centroid].

3. Apply Butterworth bandpass filtering on the windowing signal with previously obtained bands.

4. Subtract the mean of the windowing signal from the windowing signal itself to retain only the pulsatile part and remove the diffuse part.

5. Slide window over whole signal with window size = *w* and step size = *s*, which can be optimized for different datasets. For instance, in Figure 3, *w* = 14.5 s and *s* = 2 s.

6. Reconstruct the cleaned rPPG signal from the windowing segments by adding the segments.

Due to sliding windows, peaks on the edges will be smaller than the rest of the signal. This may result in peak detection issues that can be solved by multiplying both edges of the signal with coefficients (*c*), as shown in the pseudo-code (Algorithm 1) below:
**Algorithm 1:** peak amplification of the two ends of the signal*w *←* windowsize**s *←* stepsize**j *←* 0**R *←* signal****while****j≤w*/*s **do***    *c* ←
$2\frac{w}{s\left(j+1\right)}$
    *R*[s×j : $s\times \left(j+1\right)$] ← *R*[s×j : $s\times \left(j+1\right)$] × c
    *j *←* j* + 1***end while***

### 2.3. IBI Analysis

Peaks of the reconstructed signal are detected with the automatic multiscale-based peak detection (AMPD) [19] algorithm and inter-beat-intervals (IBIs) are calculated. Then, refined IBIs are calculated by removing physically impossible regions or misplaced peaks and retaining only those IBIs that satisfy the criteria below:

1. ∀*IBI* ∈ [400 ms, 1300 ms] **
2. ∀*IBI* ∈ *mean*(*IBI*) ± 0.4*mean*(*IBI*)
3. Non-overlapping window is slid over IBIs with window size 10. IBIs in each window should satisfy ∀*IBI_window_* ∈ *mean*(*IBI_window_*) ± 0.2*mean*(*IBI_window_*).

** The boundaries for the IBIs should be chosen based on the task. In this research, we estimate the HRV of adults in a seated position.

## 3. Metrics

### 3.1. HRV Metrics

**SDNN** (standard deviation of NN intervals) is a time-domain HRV metric related to the sympathetic nervous system (SNS) and parasympathetic nervous system (PNS) and associated with physical wellness such as blood pressure regulation, heart, vascular tone, and gas exchange [1]. Multiple studies show that [1,20] the range for short-term *SDNN* (<5 min) is 32–93 ms and it is given by

(4) $$SDNN=\sqrt{\frac{1}{N-1}\overset{N}{\underset{i=1}{\sum }}{\left(IBI_{i}-IBI_{mean}\right)}^{2}}$$

where *IBI* is the inter-beat interval, and *IBI_mean_* is the mean of the inter-beat interval.

**RMSSD** (root mean square of successive differences) is a time-domain HRV metric related to PNS [1] and strongly related to human productivity and energy levels. Short-term *RMSSD* (<5 min) lies within 19–75 ms [1,20]. It is given by

(5) $$RMSSD=\sqrt{\frac{1}{N-1}\overset{N-1}{\underset{i=1}{\sum }}{\left(IBI_{i+1}-IBI_{i}\right)}^{2}}$$

**Baevsky SI** (Baevsky stress index) is a stress metric that represents the mental or physical stress one is experiencing. It is very sensitive to SNS and has a range of 50 to 1000–1500 depending on stress level and stress-related illnesses [21]. It is derived using time-domain HRV metrics as follows:

(6) $$BaevskySI=\frac{AMo\left(IBI\right)}{2*Mo\left(IBI\right)*MxDMn\left(IBI\right)}$$

where *AMo*(*IBI*) is mode amplitude of IBIs, *Mo*(*IBI*) is the mode of the IBIs, and *MxDMn*(*IBI*) is the difference between the maximum and minimum IBI.

Finally, **LF/HF** (low frequency/high frequency) is a frequency-domain HRV metric that represents the balance between the PNS and the SNS [1]. It is calculated by transforming the spectral analysis of IBIs to the frequency domain with the Fast Fourier Transform (FFT). The LF [0.04–0.15 Hz] represents the SNS and the HF [0.15–0.4 Hz] represents the PNS. This is considered a metric that provides insight into the equilibrium of the autonomic nervous system and the resilience of the body to changes, stress, and anxiety [1]. LF/HF values range between 1.1 and 11.6 [1,20].

### 3.2. Evaluation Metrics

In this study, we employed several metrics to assess the performance of our proposed model. The metrics used in the study include the follofing:

**MAE** (mean absolute error) is a commonly used metric that measures the average absolute difference between predicted and actual values. *MAE* is defined as

(7) $$MAE=\frac{1}{n}\sum \left|y-\hat{y}\right|$$

where *n* is the number of data points, *y* is the actual value, and $\hat{y}$ is the predicted value.

**SD** (standard deviation) is a measure of the amount of variation or dispersion in a set of values. *SD* is defined as

(8) $$SD=sqrt(\frac{1}{n}\sum {\left(y-\hat{y}\right)}^{2})$$

where *n* is the number of data points, *y* is the actual value, and $\hat{y}$ is the predicted value.

**r** (Pearson correlation coefficient) is a measure of the linear correlation between two variables. PCC is defined as

(9) $$r=\frac{Cov\left(y,\;\hat{y}\right)}{SD\left(y\right)SD\left(\hat{y}\right)}$$

where *y* is the actual value, $\hat{y}$ is the predicted value, and *Cov*(…) is the covariance.

**The paired *t*-test** is a statistical test that compares the means of two related samples. In this study, the paired *t*-test was used to evaluate the significance of the differences between our model’s predicted values and ground truth values. The paired *t*-test is defined as

(10) $$t=\frac{\left(d-0\right)}{\left(\frac{SDd}{sqrt\left(n\right)}\right)}$$

where *d* is the mean of the differences between the predicted and actual values, 0 is the null hypothesis value, *SDd* is the standard deviation of the differences, and *n* is the number of data points.

## 4. Dataset

To validate the algorithm, we used our private dataset (Stroop) and three publicly available datasets. The summary of these datasets is shown in Table 1.

### 4.1. Stroop Dataset

Fourteen adults of ages ranging from 18 to 33 and with varying skin tones took part in our experiment. Informed consent was obtained from all subjects. Each subject was seated one meter in front of a Logitech Brio camera that recorded video at 60 fps in ambient room lighting. A CONTEC CMS-60C pulse oximeter set at a frequency of 60 Hz was used to record the ground truth PPG signal. The Stroop test [25] was used to induce cognitive stress and allow for HRV measurement under different experimental stages. In the Stroop test, participants are presented with a series of trials, where each trial consists of a color name, such as “red,” “blue,” and “green” printed in a certain ink color that may or may not match the word itself. The task requires the participant to identify the ink color while ignoring the word itself within a short span of time. The test consisted of three parts: the Rest Stage (1 min), the Stroop test with sound stimulus (3 min), and the Stroop test without sound stimulus (3 min). Subjects were allowed to relax for 2 min between each part. During the Stroop test with sound stimulus, participants heard a pleasant or irritating audio sound depending on whether they gave the correct answer.

### 4.2. Publicly Available Datasets

**UBFC rPPG [22]** consists of 42 subjects and 42 videos. Each video is approximately a minute long, 30 fps, and uncompressed. Videos are recorded in uniform, ambient lighting, and subjects play math tests to induce stress and increase heart rate.

**VIPL-HR [23]** consists of 107 subjects and 2378 videos. Video lengths range from 10 s to 1 min. Videos are compressed and recorded by three different devices. Subjects are recorded under seven different scenarios: stable scenario, talking scenario, large head movements, dark lighting, bright lighting, long distance scenario, and after exercise. In this paper, we only used videos that are longer than 16 s because it is difficult to obtain meaningful HRV results based on measurements that are less than 15 s. The number of selected videos is 1968.

**MAHNOB-HCI [24]** consists of 27 subjects and 3465 videos. To induce different emotions and feelings of stress, subjects watch different videos while sitting in front of the camera. Videos are compressed and range from 5 s to 3.5 min. In this dataset as well, only videos that are longer than 16 s are selected. The number of selected videos is 1095.

### 4.3. Dataset Preprocessing

**Figure 5:**
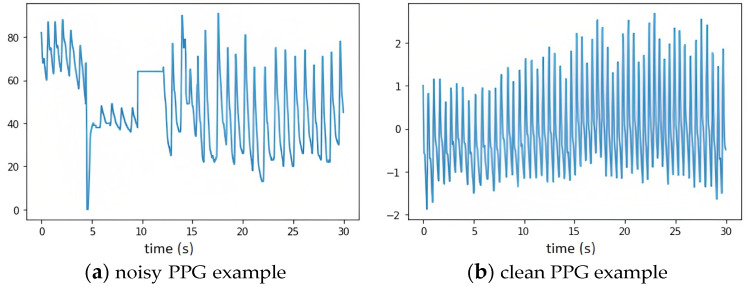
Example of noisy and clean PPG signals.

HRV is a sensitive biomarker and even a slight disturbance during the data collection process can alter the outcome dramatically. This paper [26] shows the impact of false peaks on HRV measurement and points out that if a small percentage of peaks are dislocated, HRV results will be significantly different. Therefore, noisy ground truth data must be preprocessed before being used as a benchmark to compare with camera HRV. There are several reasons why ground truth data is noisy: disconnection of the ground truth device, poor connection of electrodes with the body, body motion during data collection, slight motion of the fingers inside a pulse oximeter, etc. Examples of a noisy and clean PPG signal are shown in Figure 5. To filter out these noisy ground truth data, we came up with criteria based on biological restrictions and data analysis. First, since we are calculating HRV from the face, any obstacle between the face and the camera leads to discontinuity in the signal. Therefore, this type of data is discarded. Second, if the measured heart rate is beyond physiological and biological limits at any point, then the subject is disconnected from the ground truth data-collecting device. This kind of ground truth data cannot be used as a reference. Third, van Gent et al. [26] demonstrate that false peaks change HRV results significantly and that removing them is an optimal solution. They suggest removing *IBIs* that are off by 30% from the *meanIBI* of the chosen segment. Finally, this paper [1] reports results of more than 20 studies concluding that short-term SDNN and RMSSD (<5 min) should be less than 92 ms and 75 ms respectively. Contact-based PPG and ECG HRV results that are beyond the physiologically possible region should be removed. These criteria can be summarized as follows:

1. Remove data with a covered face at any instant in time
2. Remove data that have *HR* ∉ [45, 200]
3. Remove *IBI* ∉ *mean*(*IBI_segment_*) ± 0.3*mean*(*IBI_segment_*), where *segment* is 20–30 IBIs
4. Remove data that have *SDNN* > 100 ms or *RMSSD* > 100 ms

## 5. Results

### 5.1. Benchmarking WaveHRV

We reported the results of WaveHRV on publicly available datasets in Table 2 for SDNN and Table 3 for RMSSD. All other algorithms except FaceRPPG reported their results on the UBFC rPPG dataset only. It can be seen from Table 2 and Table 3 that WaveHRV outperformed all other methods by a significant margin except FaceRPPG RMSSD in UBFC rPPG dataset. However, it should be noted that all FaceRPPG results are benchmarked against the cleaned and filtered version of datasets. Furthermore, we observed that VIPL-HR and MAHNOB-HCI have large MAEs and even larger standard deviations.

### 5.2. WaveHRV on the Preprocessed Datasets

After filtering out noisy ground truth data according to the criteria mentioned in Section 4.3, we secure the results presented in Table 4. When comparing the results of Table 4 against Table 2 and Table 3, we see that the proposed ground truth preprocessing method performed well. MAE of SDNN of UBFC rPPG decreased from 10.5 ms to 6.15 ms, whereas RMSSD decreased from 16 ms to 10.46 ms. The effect of the proposed criteria is very noticeable on MAHNOB-HCI and VIPL-HR. By looking at the tables, we can see that the SDNN MAE of VIPL-HR decreased from 29 ms to 13.3 ms, and RMSSD MAE of VIPL-HR decreased from 41 ms to 15.1 ms. As for MAHNOB-HCI, SDNN MAE decreased from 69 ms to 17.5 ms, while RMSSD MAE decreased from 93 ms to 21.5 ms. When we look at the SD of VIPL-HR and MAHNOB-HCI, we see that the SD of VIPL-HR decreased from 45 ms to 11.1 ms for SDNN and from 70 ms to 13.1 ms for RMSSD. The SD of MAHNOB-HCI decreased from 234 ms to 12.5 ms for SDNN and from 317 ms to 14.5 ms for RMSSD.

**Figure 6:**
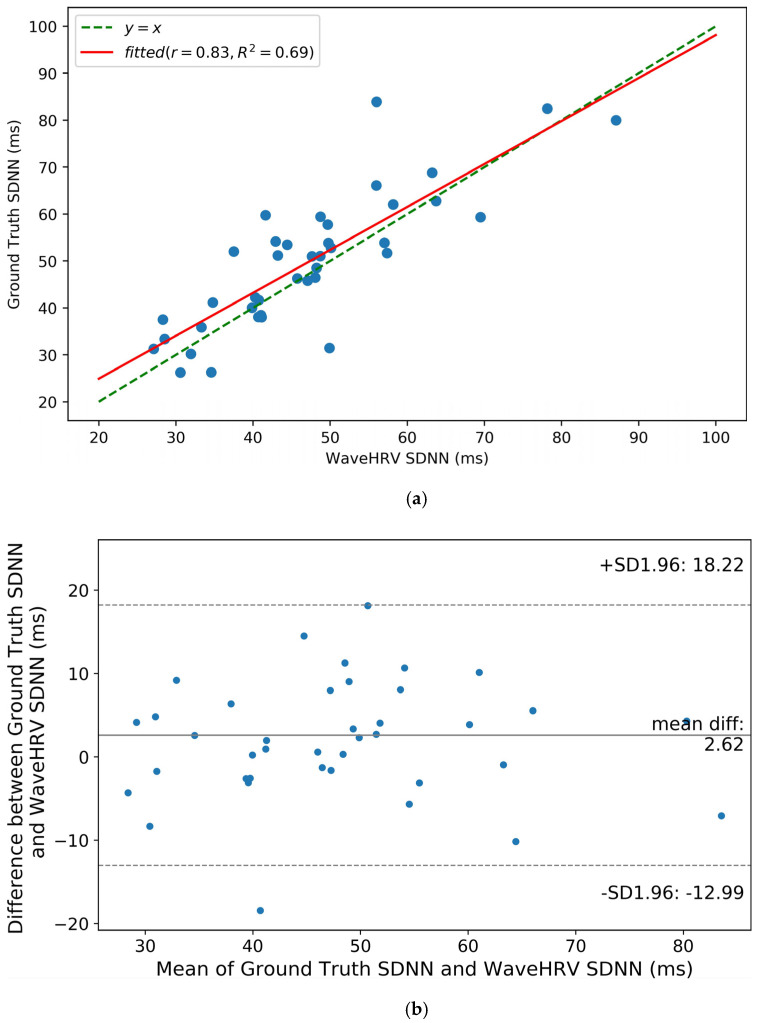
SDNN correlation (**a**) and Bland-Altman (**b**) plots for WaveHRV compared with the ground truth PPG device on the preprocessed UBFC rPPG dataset. A 95% confidence interval is marked (in ms) in the Blant-Altman plot. Pearson correlation coefficient (*r*) and coefficient of determination (*R*^2^) are given in correlation plot.

**Figure 7:**
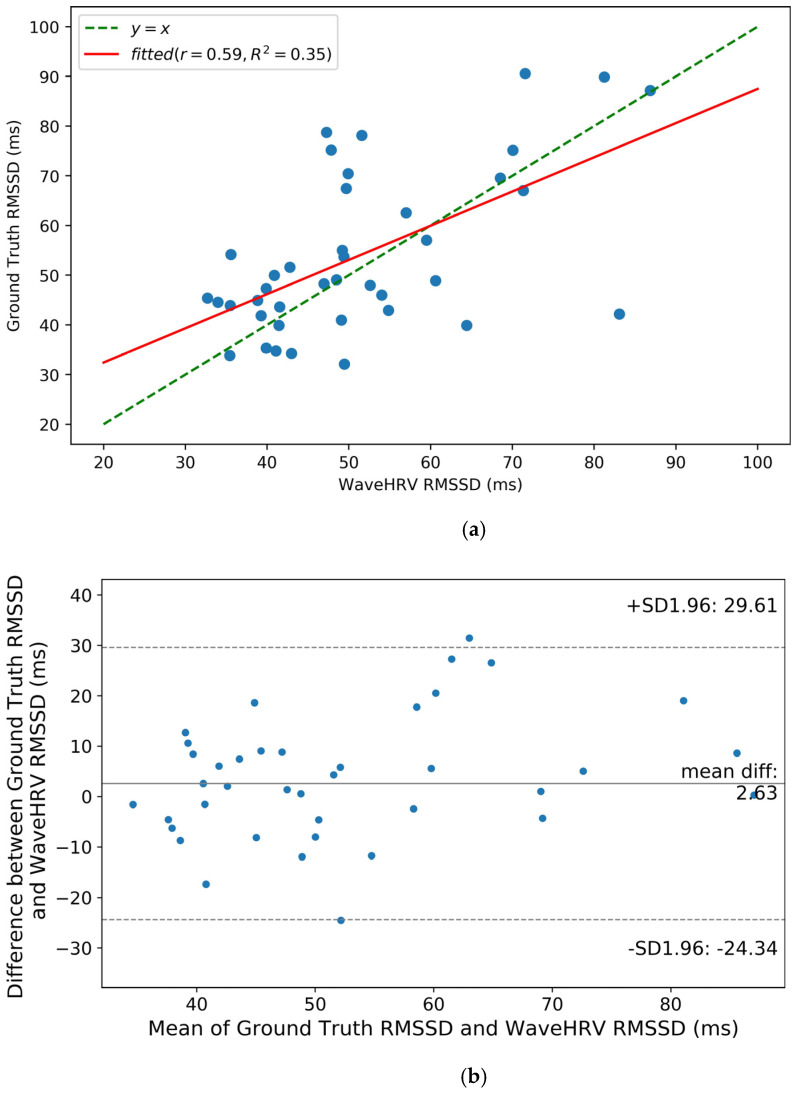
RMSSD correlation (**a**) and Bland-Altman (**b**) plots for WaveHRV compared with the ground truth PPG device on the preprocessed UBFC rPPG dataset. A 95% confidence interval is marked (in ms) in the Blant-Altman plot. Pearson correlation coefficient (*r*) and coefficient of determination (*R*^2^) are given in correlation plot.

**Figure 8:**
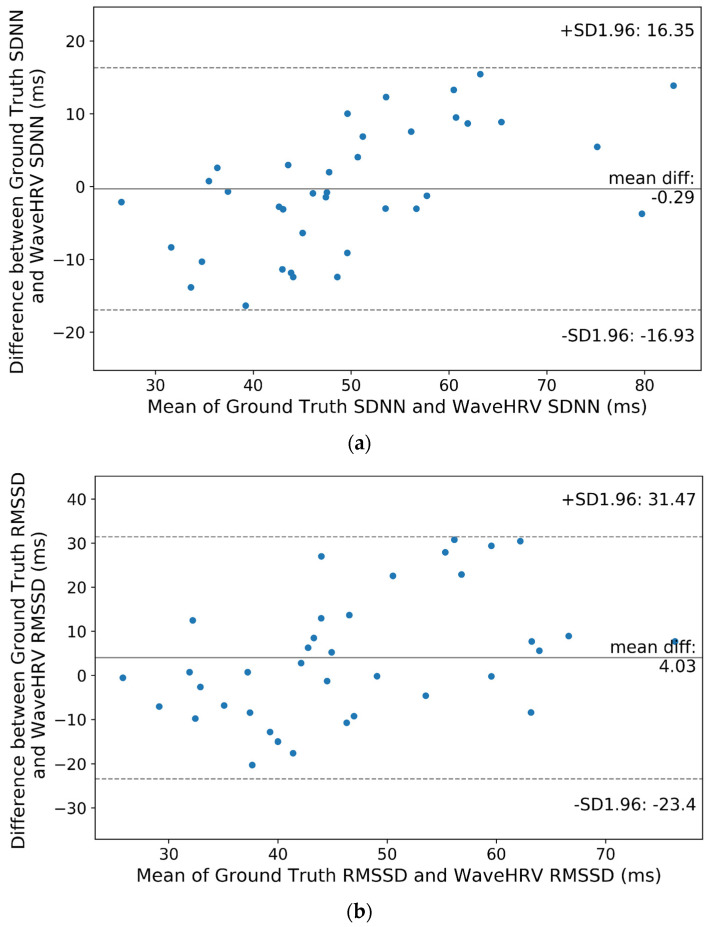
Bland-Altman plots for WaveHRV compared with the ground truth PPG device on the preprocessed Stroop dataset: (**a**) SDNN and (**b**) RMSSD. The 95% confidence intervals are marked (in ms).

**Figure 9:**
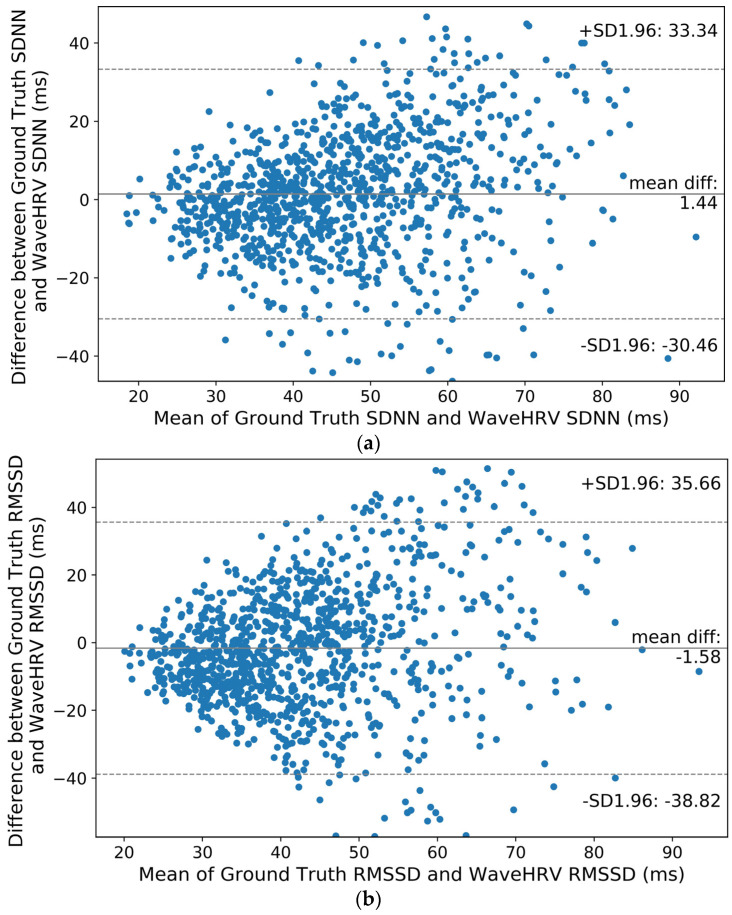
Bland-Altman plots for WaveHRV compared with the ground truth PPG device on the preprocessed VIPL-HR dataset: (**a**) SDNN and (**b**) RMSSD. The 95% confidence intervals are marked (in ms).

Bland-Altman plots of SDNN and RMSSD of three preprocessed datasets namely UBFC rPPG, VIPL-HR, and Stroop are shown in Figure 6b, Figure 7b, Figure 8, and Figure 9, respectively. It can be noticed from Figure 6b that for the UBFC rPPG dataset, the mean difference between ground truth and WaveHRV SDNN is 2.62 ms, and the paired *t*-test *p*-value = 0.05. Similarly, hypothesis testing for RMSSD gives *p*-value = 0.24. This implies that for a 95% confidence interval (CI), the average WaveHRV SDNN and RMSSD are similar or equal to the average ground truth SDNN and RMSSD. Correlation plots of SDNN and RMSSD for preprocessed UBFC rPPG are demonstrated in Figure 6a and Figure 7a. It can be noted that the Pearson correlation coefficients between WaveHRV and ground truth are 0.83 and 0.59 for SDNN and RMSSD, respectively.

Stoop dataset results (Figure 8) indicate that SDNN mean difference between WaveHRV and ground truth is −0.29 ms, whereas the RMSSD mean difference is 4.03 ms. Hypothesis testing between contact and camera HRV gives *p*-values of 0.83 and 0.09 for SDNN and RMSSD respectively. It means that at a 95% CI, average WaveHRV SDNN and RMSSD are not different from ground truth SDNN and RMSSD.

Furthermore, looking into Bland-Altman plots of the VIPL-HR dataset in Figure 9, it can be observed that SDNN mean error is 1.44 ms (*p*-value = 0.02) and RMSSD −1.58 ms (*p*-value = 0.06). Paired *t*-test reveals that at 95% CI mean WaveHRV SDNN is different from the mean ground truth SDNN, while the mean WaveHRV RMSSD is equal to the mean ground truth RMSSD. Finally, MAHNOB-HCI has SDNN −2.72 ms mean error and RMSSD −8.5 ms mean error corresponding to *p*-values = 0.02 and 10^−4^ respectively. Statistical Analysis at a 95% Confidence Interval implies that average WaveHRV SDNN and RMSSD are different from average ground truth SDNN and RMSSD.

### 5.3. Stress Measurement

The performance of WaveHRV on physiological stress-related metrics is given in Table 5. To get better frequency resolution in frequency-domain metrics, videos that are longer than 30 s are considered in this part. LF/HF is a metric of homeostasis and resilience of the autonomous nervous system (ANS) to stress and anxiety. LF/HF values range between 1–11.5 and Table 5 illustrates that LF/HF MAEs lie between 0.26–0.67. Therefore, WaveHRV could be used to obtain LF/HF and has the potential to offer insights into the balance and equilibrium of ANS.

The Baevsky stress index (BaevskySI), also known as the strain index, characterizes a person’s sympathetic nervous system activity (SNS) and is a good indicator of physical and mental load. Table 5 reveals that the MAE of BaevskySI from the contact-based device and WaveHRV is within 40–60 for UBFC rPPG, Stroop, and MAHNOB-HCI datasets, while VIPL-HR has BaevskySI MAE ≈ 100. As mentioned above in Section 3.1, BaevskySI has values between 50–1500, and looking at the results of WaveHRV, it can be inferred that our algorithm can be utilized to categorize and identify different stress levels.

## 6. Discussion

It has been revealed that both the MAE and SD of VIPL-HR and MAHNOB-HCI datasets have significantly dropped after the implementation of the data preprocessing step mentioned in Section 4. The primary reason for this phenomenon is caused by disconnected or poorly connected electrodes and pulse oximeters, slight motion of fingers inside pulse oximeters, and motion during data collection.

Furthermore, from Table 4, we note that WaveHRV has lower MAEs on UBFC rPPG and Stroop datasets than on challenging datasets like VIPL-HR and MAHNOB-HCI. UBFC rPPG and Stroop are not compressed and have uniform ambient light, whereas VIPL-HR and MAHNOB-HCI are compressed and recorded under non-uniform or dim lighting. Moreover, in some scenarios of the VIPL-HR, subjects perform large head movements, talk, or are sited further away from the camera.

Similar conclusions can be attained from statistical analyses and Bland-Altman plots: when the subjects are not under frequent motion and in a well-lit environment like UBFC rPPG and Stroop datasets, the average WaveHRV SDNN and RMSSD are similar to ground truth SDNN and RMSSD. However, for more challenging, real-life scenarios where there is significant motion and poor lighting conditions like VIPL-HR and MAHNOB-HCI, mean WaveHRV results are different from mean ground truth results.

## 7. Conclusions

In this paper, we have presented WaveHRV, a novel algorithm for HRV extraction from a portable camera. We benchmarked our algorithm against other methods and demonstrated that WaveHRV outperforms other methods on publicly available datasets. Furthermore, we presented a straightforward yet powerful technique to clean ground truth data and highlighted its performance. We also demonstrated the potential for an off-shelf camera to measure stress and mental well-being via the Baevsky stress index. A further direction for this research would include the improvement of HRV algorithms under challenging scenarios such as large head movements and dim lighting to reduce the discrepancy between camera HRV and contact HRV. In addition, work could examine the relationship between HRV and different stress, energy, and productivity metrics.

## Figures and Tables

**Table 1:** Summary of datasets used in this paper.

Dataset	# Videos	Fps	Resolution	Compressed	Ground Truth
Stroop	42	60	640 × 480	no	PPG (60 Hz)
UBFC rPPG [22]	42	30	640 × 480	no	PPG (30 Hz)
VIPL-HR [23]	1968	25/30	1920 × 1080	yes	PPG (60 Hz)
MAHNOB-HCI [24]	1095	60	780 × 580	yes	ECG (256 Hz)

**Table 2:** Performance of SDNN measurement for the UBFC rPPG, VPIL-HR, and MAHNOB-HCI datasets. Superior performance is highlighted in green.

Dataset	UBFC rPPG	VIPL-HR	MAHNOB-HCI
	MAE ± SD (ms)	MAE ± SD (ms)	MAE ± SD (ms)
WaveHRV	10.5 ± 7.9	29 ± 45	69 ± 234
FaceRPPG * [5]	19 ± 14.5	49 ± 45	107 ± 51
SSF [2]	25	-	-
PulseGAN [13]	24.3	-	-

* The results of this method are given against a cleaned version of the data.

**Table 3:** Performance of RMSSD measurement for the UBFC rPPG, VPIL-HR, and MAHNOB-HCI datasets. Superior performance is highlighted in green.

Dataset	UBFC rPPG	VIPL-HR	MAHNOB-HCI
	MAE ± SD (ms)	MAE ± SD (ms)	MAE ± SD (ms)
WaveHRV	16 ± 14	41 ± 70	93 ± 317
FaceRPPG * [5]	16 ± 22.5	73 ± 57.8	108 ± 51
SSF [2]	47	-	-

* The results of this method are given against a cleaned version of the data.

**Table 4:** SDNN and RMSSD performance of WaveHRV on Preprocessed Datasets.

Dataset	Stroop	UBFC rPPG	VIPL-HR	MAHNOB-HCI
	MAE ± SD	MAE ± SD	MAE ± SD	MAE ± SD
SDNN (ms)	7.0 ± 4.80	6.15 ± 5.69	13.3 ± 11.1	17.5 ± 12.5
RMSSD (ms)	11.35 ± 9.13	10.46 ± 9.32	15.1 ± 13.1	21.5 ± 14.5

**Table 5:** Baevsky SI and LF/HF performance of WaveHRV on Preprocessed Datasets.

Dataset	Stroop	UBFC rPPG	VIPL-HR *	MAHNOB-HCI *
	MAE ± SD	MAE ± SD	MAE ± SD	MAE ± SD
BaevskySI	38 ± 45	42 ± 35	98 ± 122	55 ± 65
LF/HF	0.67 ± 0.76	0.26 ± 0.32	0.43 ± 0.63	0.33 ± 0.39

* Videos that are longer than 30 s are considered.

## Data Availability

Data sharing not applicable.
